# A sibship with duplication of Xq28 inherited from the mother; genomic characterization and clinical outcomes

**DOI:** 10.1186/s12881-017-0394-7

**Published:** 2017-03-17

**Authors:** Dong Keon Yon, Ji Eun Park, Seung Jun Kim, Sung Han Shim, Kyu Young Chae

**Affiliations:** 1 Department of Pediatrics, CHA Bundang Medical Center, School of Medicine, CHA University, 351 Yatap-dong, Bundang-gu, Seongnam, 463-712 Republic of Korea; 2 Genetics Laboratory, Fertility Center, CHA Gangnam Medical Center, School of Medicine, CHA University, 606-13 Yeoksam-dong, Gangnam-gu, Seoul, 06135 Republic of Korea; 3 GenoLifeCare Division, BioCore, Seoul, Republic of Korea; 4 Department of Biomedical Science, College of Life Science, CHA University, 335 Pankyo-ro, Bundang-gu, Seongnam, 13488 Republic of Korea

**Keywords:** MECP2, IRAK1, Xq28 duplication, MECP2 duplication syndrome

## Abstract

**Background:**

Loss-of-function mutations in methyl-CpG-binding protein 2 (*MECP2*; MIM *300005) results in the Rett syndrome, whereas gain-of-function mutations are associated with the *MECP2* duplication syndrome.

**Methods:**

We did research on a family with two brothers showing Xq28 duplication syndrome using various molecular cytogenetic techniques such as multiplex ligation-dependent probe amplification and array-based genomic hybridization.

**Results:**

The duplicated region had several genes including *MECP2* and interleukin-1 receptor associated kinase 1 (*IRAK1*; MIM *300283). *MECP2* and *IRAK1* were associated with the neurological phenotypes in dose-sensitive and dose-critical manner. The brothers demonstrated severe intellectual disability, autistic features, generalized hypotonia, recurrent infections, epilepsy, choreiform movements such as hand-wringing movement, and moderate increased spasticity with the lower limbs. The X-inactivation test showed a complete skewed X inactivation pattern of mother. In this reason, the mother had the same loci duplication but showed significantly little neurological manifestation compared to the two sons.

**Conclusions:**

*MECP2*/*IRAK1* duplication at Xq28 is inherited as an X-linked recessive trait and male-specific disorder associated with severe intellectual disability. We tried to analyze the information of the relationship between neuropsychiatric phenotype and the extent of duplication at Xq28 by comparing with previous reports.

**Electronic supplementary material:**

The online version of this article (doi:10.1186/s12881-017-0394-7) contains supplementary material, which is available to authorized users.

## Background

X-linked intellectual disability (XLID) is found in approximately 5–10% of the intellectually disabled men [1]. XLID is divided into syndromic forms in which intellectual disability is one of many symptoms, and non-syndromic forms, in which intellectual disability is the only symptom. In a previous study, 102 genes were shown to be associated with 81 out of 160 cases of XLID syndromes in over 50 families with non-syndromic XLID [1]. Most of the genetic defects leading to XLID are loss-of-function mutations, including point mutations (frame shift, nonsense, and missense), microdeletions, and translocations. XLID caused by gain-of-function is via microduplication where causal genes are abnormally copied. A well-known XLID microduplication region is Xq28, in which there is duplication of X-linked genes such as methyl-CpG-binding protein 2 (*MECP2*; MIM *300005) a key gene involved in Rett syndrome, a neurodevelopmental disorder that affects mostly girls Xq28 duplication syndrome is an important aspect of XLID. Mutations in the *MECP2* gene in Xq28 were first reported in patients with Rett syndrome in 1999 [2] and other X-linked severe neurodevelopmental disorders. Particularly, large deletions, single base mutations, or small frame shift mutations in *MECP2* are mostly commonly associated with Rett syndrome [3, 4]. Classical Rett syndrome is typically characterized by a period of normal development until 6 to 18 months of age. After this age, motor and speak skills regress, followed by loss of useful hand skills; rather afflicted children exhibit repetitive hand motions, such as hand wringing. Additional symptoms of Rett syndrome include acquired microcephaly, profound intellectual disability, epilepsy, ataxia, and autistic behaviors. There is a wide spectrum of disease severity for Rett syndrome as determined by molecular diagnostics [5].

While loss-of-function mutations in *MECP2* result in Rett syndrome gain-of-function mutations are associated with *MECP2* duplication syndrome. *MECP2* duplications were initially described in a female patient presenting with a speech variant symptom and was diagnosed molecularly with atypical Rett syndrome [6]. *MECP2* duplication syndrome and Rett syndrome share overlapping clinical phenotypes include intellectual disability, motor deficits, epilepsy, hypotonia, and progressive spasticity [7, 8]. Males with Xq28 duplication, including duplications at the *MECP2* locus spanning 0.3–4 Mb, have subsequently been reported by employing the multiplex ligation-dependent probe amplification (MLPA) and array-based comparative genomic hybridization (array-CGH) [7, 9–11]. Common phenotypes in males with *MECP2* duplication are severe to profound X-linked intellectual disability, Rett syndromic features, progressive spasticity, neonatal or infantile hypotonia, poor speech development, recurrent respiratory infections, epilepsy, and dysmorphic facial features such as large ears, mid-face hypoplasia, brachycephaly, and depressed nasal bridge [8].

In this study we identified one family with two brothers with Xq28 duplication syndrome and a mother who was a carrier with the same loci duplication. We characterized the duplicated region of the brothers and the mother using various molecular and cytogenetic techniques and to present clinical features.

## Methods

### Patients

**Fig. 1:**
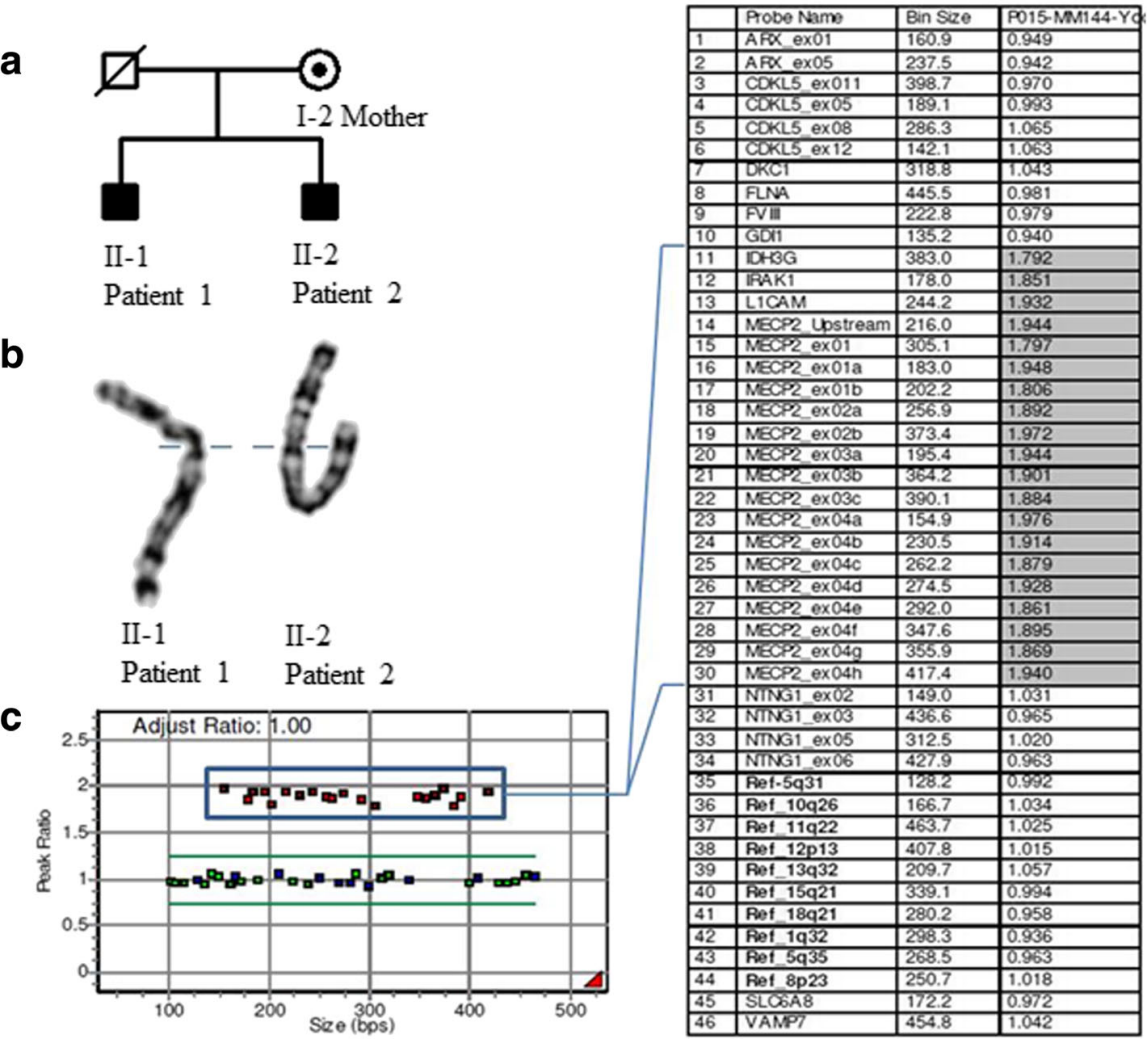
A pedigree of the family and the results of MLPA analysis. **a** A pedigree of the family. **b** Apparently normal GTG-banded X chromosomes of the patients. **c** MLPA result of the II-1 patient 1. MLPA analysis using the P015 MECP probemix showed duplications of the *MECP2* and several other genes (rectangle)

A family including two sons aged 11 and 10 years old and their mother with Xq28 duplication was researched (Fig. 1a and Additional file 1: Figure S1). A duplication of 411.478kb in Xq28 was identified in all three family members using the MLPA and array-CGH.

### Patient 1

Patient 1 was an 11-year-old boy who was born at 41 weeks’ gestation by spontaneous vaginal delivery. At birth the boy weighed 2500 g, and there was no history of brain damage (Table 1). The father passed away in car accident, and the mother was healthy and non-consanguineous. At birth, he was treated in the neonatal intensive care unit over three weeks due to infantile central hypotonia with poor sucking. His feeding problems in infancy needed to be remedied via special feeding techniques and improved gradually with age. His developmental delay became more obvious as time went by. Independent walking started at 36 months of age. He was vulnerable to bacterial infections such as pneumonia and gastroenteritis, requiring hospitalization about three times a year. Currently, he cannot make eye contact and social-emotional reciprocity, nor form any meaningful words. And he have excessive adherence to the pencil, hand stereotypes including hand shaking and hyper-reactivity to unusual interest. He can not live a social life without a carer.

**Table 1:** Clinical characteristics of the patients

Patient	Patient 1	Patient 2	Mother
Age	11 years	10 years	38 years
Gender	Male	Male	Female
Prenatal history	41 weeks, 2500g, NSVD Infantile hypotonia	41 weeks, 3000g, C/sec	
Gross motor of K-CDR	30 month function	30 month function	
Fine motor of K-CDR	20 month function	22 month function	
Self-help level of K-CDR	24 month function	12 month function	
Social level of K-CDR	18 month function	16 month function	
Language development of K-CDR	14 month function	14 month function	
Mental scale of BSID II	24 month function	9 month function	
Motor scale of BSID II	14 month function	12 month function	
K-WAIS-IV			77 (6th percentile)
Head and face	Brachycephaly Large & Low set ears Nose with upturned nares	Midface hypoplasia Large ears Low nasal bridge	Prominent lips
Autism or autistic features	O	O	X
Generalized hypotonia	O	O	X
Choreiform movements	O	O	X
Progressice spasticity	O	O	X
Drooling	O	O	X
Bruxism	O	X	X
Brain-MRI finging	normal	Normal	None
Recurrent infecition	Pneumonia and gastroenteritis	Pneumonia	None
Medical problem	Epilepsy treated with oxycarbamazepine	Epilepsy treated with valproate	Narcolepsy treated with modafinil and SSRI

*BSID II* Bayley Scales of Infant Development test II, *C/sec* Cesarean section, *K-CDR* Korean-Child Development Review, *K-WAIS-IV* Korean Wechsler Adult Intelligence Scale fourth edition, *NSVD* normal spontaneous vaginal delivery, *SSRI* selective serotonin reuptake inhibitor

**Fig. 2:**
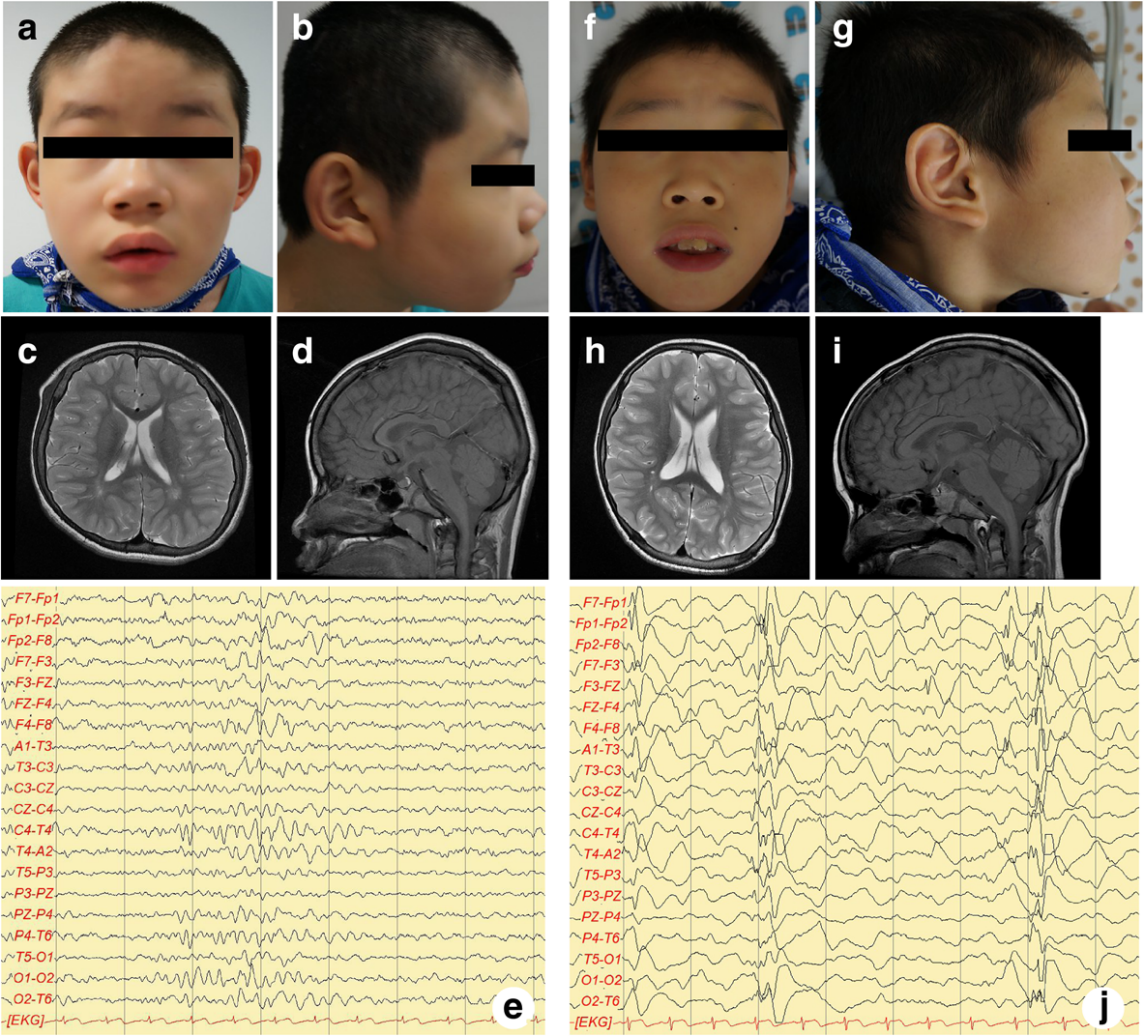
Clinical features of patient 1 and 2. Clinical features of patient 1 (**a**-**e**). Facial dysmorphisms with brachycephaly, slightly upturned nares, large and low set ears were observed (**a**, **b**). Axial T2 (**c**) and sagital T1 (**d**)-weighted brain MRI images were in normal limits. interictal EEG shows epileptic sharp wave discharges from the right temporal cerebral area with poorly regulated posterior rhythm and slow background activity (**e**). Clinical features of patient 2 (**f**-**j**). Facial dysmorphisms with large ears, slightly upturned nares, and midface hypoplasia followed by depressed nasal bridge were observed (**f**, **g**). Axial T2 (**h**) and sagital T1 (**i**)-weighted brain MRI images showed no abnormalities. interictal-EEG reveals frequent generalized burst of epileptic sharp wave discharges from the both frontal cerebral area followed by attenuation of background activity (**j**)

At the time of study evaluation his height was 149 cm (50–75^th^ percentile), weight was 50 kg (75–90^th^ percentile), and occipito-frontal circumference was 53 cm (75–90^th^ percentile). According to the Korean-Child Development Review (K-CDR) [12], his gross and fine motor skills corresponded to those observed in children aged 30 and 20 months, respectively. Self-help and social levels were equivalent to that of a 24- and 18-month-old, respectively. Language development was at the 14-months level. He was diagnosed with autistic spectrum disorder in accordance with the fifth edition of the Diagnostic and Statistical Manual of Mental Disorders (DSM-V) [13]. According to the mental developmental index of the Bayley Scales of Infant Development test II (BSID II) [14], the equivalent age of the patient was 24 months, implying severe intellectual disability. Mild dysmorphic features (brachycephaly, slightly upturned nares, and large and low set ears) and generalized hypotonia were observed (Fig. 2a-d). Upon neurological examination, he had significant weakness in the extremities; based on the Medical Research Council (MRC) for muscle strength the patient was a grade 3 with regards to choreiform gait movements, such as hand wringing, and moderate to increased spasticity of the lower limbs.

He presented with generalized tonic-clonic type seizures at 10 years of age. In his interictal-electroencephalogram (EEG) there were epileptic sharp wave discharges from the right temporal cerebral area with poorly regulated posterior rhythm and slow background activity (Fig. 2e). Magnetic resonance imaging (MRI) scan of the brain revealed no abnormalities. At 4-year follow-up, seizures were controlled via oxcarbazepine administration without any side effects.

### Patient 2

Patient 2 was Patient 1’s younger brother. He was 10 years old and showed severe autistic features along with global developmental delay and intellectual disability. He was born at 41 weeks’ gestation via cesarean section (due to cephalopelvic disproportion) with a birth weight of 3000 g. At the time of birth there was no history of brain damage. Delayed development was apparent, as he was unable to walk independently before 25 months of age. He has had multiple bacterial infections resulting in pneumonia, each time leading to hospitalization. He was diagnosed with autistic spectrum disorder in the DSM-V [14] at 8 years of age, and he has been rehabilitated ever since.

At the time of the study his height was 139 cm (75–90^th^ percentile), weight was 37 kg, and head circumference was 54 cm (75–90^th^ percentile). According to the K-CDR scale [12], the boy’s gross and fine motor skills correlated to 30 and 22 months, respectively. His self-help, social, and language skills corresponded to 12, 16, and 14 months of age, respectively. According to the mental developmental index of the BSID II [14], the age equivalent of the patient was 9 months, suggesting severe intellectual disability. Upon physical examination, mild dysmorphic features (large ears, slightly upturned nares, and mid-face hypoplasia followed by a depressed nasal bridge) and generalized hypotonia were noted (Fig. 2f–i). Upon neurological examination, the profiles of Patient 1 and Patient 2 were similar such as decreased MRC scale grade 3 with regards to choreiform gait movements and moderate to increased spasticity of the lower limbs.

Generalized tonic-clonic type seizures started at the age of 10 years. In his interictal-EEG there were frequent generalized bursts of epileptic sharp wave discharges from both the frontal cerebral area followed by attenuation of background activity (Fig. 2j). MRI of the brain showed no abnormalities. He was initially treated with valproic acid in order to treat his seizures. And seizures were well controlled without any side effects for 4 years.

He has no evidence of complement deficiency 10 years old (C3: 131 C4: 16.50, and CH50: 50 mg/dL). Immunoglobulin (Ig) levels and IgG subclass were normal (IgM: 228, IgG2: 279, IgG3: 82, and IgG4: 10 mg/dL and IgE: 21.2 U/mL) or mildly elevated (IgG: 1970, IgA: 554, and IgG1: 1347 mg/dL). The anti-nuclear antibody (ANA) was negative.

Currently he cannot make eye contact and facial expressions, and is unable to produce any meaningful words. He has a strong attachment to unusual objects such as flipping through pages of books, and he drools excessively. He is followed with regular checkup for 4 years without seizure.

### Mother

The 38-year-old mother had normal cognition but suffered from psychiatric symptoms including depressive episodes and narcolepsy with frequent cataplexy. She sometimes collapsed when standing, or walking. She took medicine to treat these symptoms including modafinil and selective serotonin reuptake inhibitor. She also experienced abnormal menstrual cycles. At the time of the study, her weight was 70 kg (Body Mass Index: 28.4, > 99^th^ percentile), and her height was 157 cm (10–25^th^ percentile). Her lips were prominent, and her OFC was 54 cm (25–50^th^ percentile). Her intelligence quotient (IQ) as measured by the Korean Wechsler Adult Intelligence Scale fourth edition (K-WAIS-IV) [6] was 77 (6^th^ percentile), suggesting borderline intellectual disability. No other abnormalities were reported.

### Cytogenetic analysis

For high-resolution chromosome analysis peripheral lymphocytes were cultured by conventional thymidine methods [15]. Cell harvests, fixations, and slide preparations were performed using a standard protocol. The slides were stained by the GTG-banding method, and 20 metaphases were examined.

### MLPA analysis

Genomic DNAs of the proband and all his family members were extracted from peripheral blood samples using the QuickGene-610L Nucleic Acid Isolation System (Fujifilm Tokyo, Japan). The extracted DNAs were measured by a NanoDrop® spectrophotometer, ND-1000 (NanoDrop Technologies, Wilmington, DE). To identify whether or not the patient had a common microdeletion, the MLPA was performed using the SALSA MLPA P245 Microdeletion Syndromes-1 probemix which contains probes for 23 different microdeletion syndromes and the SALSA MLPA P015 MECP2 probemix (MRC-Holland, Amsterdam, Netherlands). All procedures were accomplished according to the manufacturer’s protocol. The reaction products were loaded on an ABI Prism 3130XL automatic genetic analyzer (Applied Biosystems, Foster City, CA) and analyzed by the GeneMaker v1.95 software (Softgenetics, State College, PA).

### Array-CGH

To characterize the breakpoints and the size of the duplicated region array CGH using the Cytoscan® HD array (Affymetrix, Santa Clara, CA) was performed. All procedures including DNA labeling, hybridization, and post-hybridization washing were carried out by a commercial service provider (BioCore, Korea) according to the manufacturer’s protocol. The arrays were scanned and analyzed using the GeneChip® 3000 Scanner (Affymetrix, Santa Clara, CA) and the Affymetrix Chromosome Analysis Suite (ChAS) v1.2 Software, respectively.

### X-inactivation test (XCI)

X chromosome inactivation pattern of the mother was examined by the human androgen-receptor locus (*HUMARA*) methylation analysis [16]. Briefly genomic DNA was digested with methylation-sensitive restriction endonuclease, *HpaII* and then the polymorphic CAG repeated in the *HUMARA* locus was amplified by polymerase chain reaction (PCR). Skewed XCI was determined by the PCR product size and the inherited pattern of the alleles.

## Results

High-resolution cytogenetic analysis showed apparently normal karyotypes in the proband and family members. However the MLPA analysis used the P245 microdeletion syndromes-1 probemix detected a duplication of the *MECP2* region in the proband, his older brother and mother. These results were re-evaluated using the P015 *MECP2* probemix and in addition to *MECP2* gene, duplication signals for *IRAK1*, *L1CAM* and *IDH3G* were also identified (Fig. 1b and c).

**Fig. 3:**
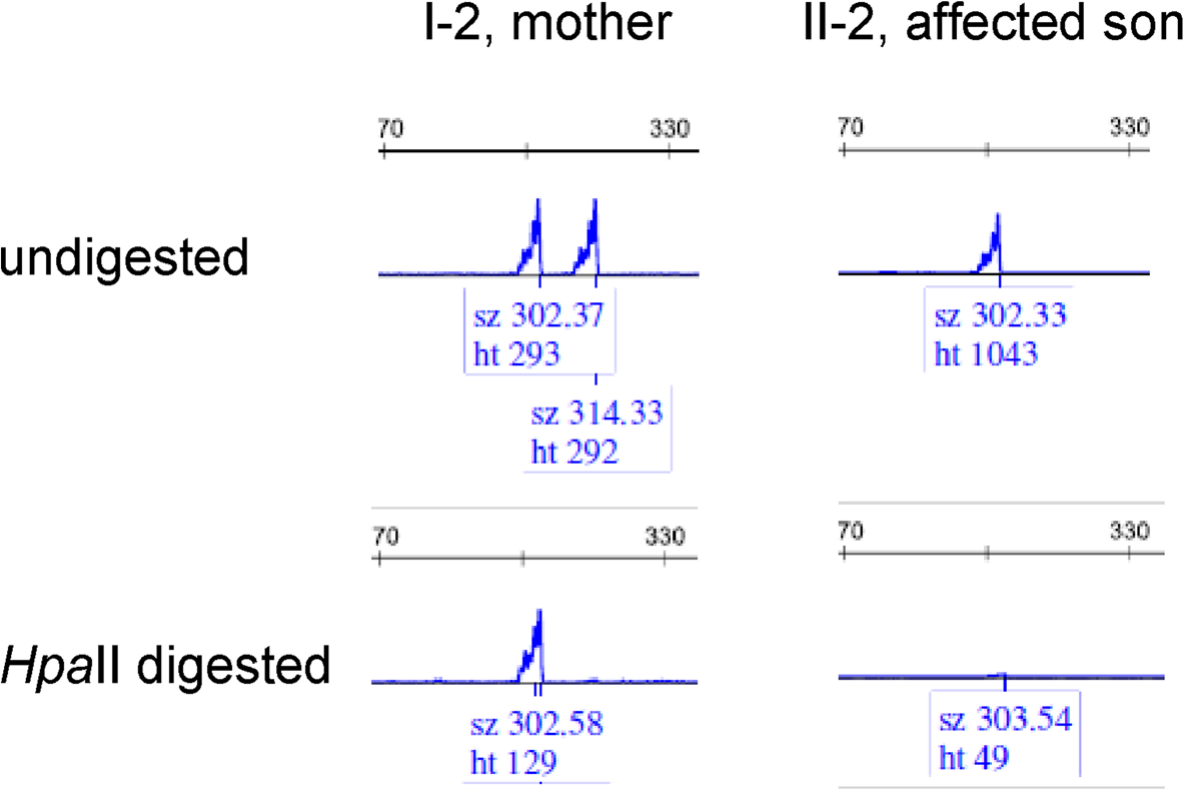
X-inactivation analysis. (Top) The mother (I-2) is a heterozygous for HUMARA allele (302 and 314). The affected son received the 302 allele from the mother. (Bottom) After the methylation-sensitive restriction endonuclease, HpaII digestion, the only one allele 302 (methylated, inactive) was identified in the mother. This result represented completely skewed (100:0) inactivation of the X chromosome containing the MECP2 region duplication

In the *HUMARA* analysis the mother was heterozygous (302/314) and the proband was hemizygous (302) for *HUMARA* locus. After *HpaII* digestion (only unmethylated allele digested), only one allele (302) was amplified in the mother and no amplification product was obtained in the proband. These results indicated that XCI in the mother was completely skewed, only the X chromosome with duplication of the Xq28 region were inactivated in almost all cells (Fig. 3).

Array CGH analysis confirmed the earlier MLPA results and revealed the exact breakpoints and the size of the duplicated region. A gain signal at Xq28 region spanning about 411kb was identified in both patients and their mother (chrX:153,0027,303-153,438,781). Array CGH data are summarized in table 2.

**Table 2:** Summary of array-comparative genomic hybridization results

Chromosome region	Start	End	Size (Kb)	Gain/loss	Marker count	OMIM gene
Xq28	1530027303	153438781	411.478	gain	1036	*PLXNB3, SRPK3, IDH3G, SSR4, PDZD4, L1CAM, AVPR2, ARHGAP4, NAA10, RENBP, HCFC1, TMEM187, MIR3202-1, MIR3202-2, IRAK1, MIR718, MECP2, OPN1LW*

## Discussion

A duplication of 411.478 kb in Xq28 including the *MECP2* and *IRAK1* genes, was identified in our study, while varying sizes of 200–4000 kb have been reported in other studies [10, 11, 17].

Xq28 duplication syndrome involving *MECP2* gene is inherited as an X-linked recessive trait associated with severe to profound intellectual disability. Clinical manifestations are male-specific with symptoms such as recurrent infections that may lead to premature death, infantile hypotonia, mild dysmorphic features, progressive spasticity of the lower limbs observed after 3 years of age, autistic features, and generalized epilepsy [8]. These clinical features were first described as Lubs-type X-linked mental retardation syndrome (MIM #300260) [18], and current understanding of the genetic basis was recognized as chromosomal duplication of the *MECP2* region [19]. The function of MeCP2 as repressor or activator of transcription, chromatin architecture, and regulation of RNA splicing contributes to essential brain development as a regulator of synaptic and neuronal plasticity [20, 21]. M*ECP2* overexpression in mice also present as a neurological phenotype. The mice exhibited hallmark symptoms such as abnormal forepaw movements, hypoactivity, and premature death [22]. Additionally, mice that overexpressed wild-type human *MECP2* from its own promoter developed progressive seizures [22]. Interestingly enough, this study and additional scans of the literature indicate that epilepsy associated with *MECP2* duplication syndrome cannot be considered as a useful marker for early diagnosis. However, epilepsy is present in >90% of adolescent patients and shows a peculiar electro-clinical pattern. In a recent review, Ramocki et al. reported in >50% incidence of epilepsy in 110 patients with *MECP2* duplication, resulting in severe seizures of multiple types [8]. More than half of the patients were resistant to seizure drug medicine, and there was also a case in which a patient was on a combination of multiple seizure drugs, and a ketogenic diet [23]. The common interictal EEG pattern in patients with *MECP2* duplication from this study revealed asynchronous waves and generalized discharge of spikes from the fronto-temporal area with abnormal EEG background frequency and rhythm, such as the abnormal K complex and sleep spindle [23]. These EEG patterns were similar to those found in other genetic syndromes, such as 4p- syndrome or Angelman syndrome [24], thus perhaps making diagnosis difficult. With regards to possible therapies, a previous study reported that deep brain stimulation reduced convulsions up to 65% in case of failed multiple anti-epileptic drugs [25]. Epilepsy is a significant sign of *MECP2* duplication syndrome, and an EEG follow-up of these patients from early childhood should be encouraged. Moreover, the definition of a more specific epileptic phenotype could be useful in order to suspect *MECP2* duplication syndrome in older, undiagnosed patients. In our study, Patient 1 was treated with oxcarbazepine and Patient 2 with valproic acid, and serial follow-up interictal EEGs are needed to confirm the clinical improvement.

*MECP2* severity is linked to copy number. Del Gaudio et al*.* found a *MECP2* triplication in one boy resulting in a more severe phenotype than MECP2 duplication [9]. This implies that copy number is associated with enhanced disease. Thus, the *MECP2* gene is a dose-sensitive and a critical gene in neurological development and disorders. Additionally in our study, we note that among the dysmorphic and neurological XLID phenotypes observed in Patient 1 and 2, the influence of *MECP2* was most critical in the manifestation of these features. Undiagnosed patients presenting with *MECP2* duplication phenotypes and seizure should be tested for *MECP2* duplications via the array-CGH or MLPA, diagnostics that were crucial in our study.

X-linked genetic etiologies associated with infantile central hypotonia are Pelizaeus-Merzbacher Allan-Herndon-Dudley, Coffin-Lowry, Lowe, alpha thalassemia, and *MECP2* spectrum disorders such as Rett syndrome and *MECP2* duplication at Xq28 [26]. Treatment for infantile central hypotonia is massive supportive and symptomatic care. In our study, Patient 1 was treated in the neonatal intensive care unit due to infantile central hypotonia with poor sucking. If a genetic study was carried out at that time of his birth, he could have been diagnosed. We suggest that infantile central hypotonia patients must be considered for testing for Xq28 duplication syndrome, particularly duplication at *MECP2.*

Several children with duplicated *MECP2* and *IRAK1* were reported to have suffered severe recurrent respiratory infections with decreased IgA levels and poor antibody responses due to polysaccharide antigens exhibited in some children [26]. Even though Patient 2 had severe recurrent respiratory infections requiring hospitalization thrice a year, there was no evidence of immunodeficiency as per our assessment of complement and Ig antibodies. In a previous study, patients with overexpressed *MECP2* were partially immunodeficient possibly due to the overexpression of *MECP2* suppressing interferon (IFN)-γ secretion from T helper cells [27]. The intermediate signaling molecule, *IRAK1*, is known to have a critical roles in the Toll-like receptor (TLR) signaling pathway and activation and regulation of innate and adaptive immunity [28, 29]. Thus, cellular immune dysfunction, due in part to the suppression of IFN-γ production from T helper cells, is potentially caused by overexpression of *MECP2* and *IRAK1* in Xq28 duplication syndrome. However, additional studies for humoral immune dysfunction in Xq28 duplication are required.

Females rarely display the canonical neurological phenotypes including intellectual disability, associated with *MECP2* duplication is due to extreme (>80%) or complete (100%) skewed XCI [30, 31]. The Mother of Patients 1 and 2 had the same duplication profile as her son. However, she had completely skewed (100%) XCI, including the duplicated *MECP2* region, and she significantly little neurological manifestations as compared to her sons. Female patients carrying *MECP2* mutations in Rett syndrome display X-linked dominant inheritance and not skewed XCI. However, female carriers with *MECP2* duplication exhibit X-linked recessive inheritance and skewed XCI. This implies that overexpression of the neighboring genes in the duplicated Xq28 region rather than *MECP2* itself causes negative selection, leading to XCI during the early embryonic period [32]. Mayo et al*.* specifically noted that the arrest-defective protein 1 homolog A (*ARD1A* or *NAA10*; MIM *300013) and host cell factor C1 (*HCFC1*; MIM *300019) as genes responsible for induced preferential XCI in females [33]. In our study the Mother had duplication in parts of *NAA10* and *HCFC1*, and thus the possibility of negative selection was considered. In prior studies, obvious clinical phenotypes of *MECP2* duplication syndrome was reported in females with less than 70% skewed XCI [31, 34–36]. Additional research on the mechanism of XCI is needed, especially since negative selection may not occur despite the duplication of *NAA1* and *HCFC1*. Further studies dissecting the genotype-phenotype correlation against the degree of *MECP2* expression are also required.

## Conclusions

*MECP2*/*IRAK1* duplication at Xq28 is inherited as an X-linked recessive trait and male-specific disorder associated with severe intellectual disability. *MECP2*/*IRAK1* was associated with primary dose-sensitive and dose-critical gene for the neurological phenotypes. Carrier mother had mild neuropsychiatric symptoms despite of markedly skewed XCI. Additional research is needed to know the relationship between neuropsychiatric phenotype and degree of MECP2 expression.

## Additional file

Additional file 1:
**Figure S1.** A pedigree of the family and the results of MLPA analysis. (TIF 626 kb)

## Abbreviations

ANA: Anti-nuclear antibody
ARD1A or NAA10: Arrest-defective protein 1 homolog A
Array-CGH: Array-based genomic hybridization
BSID II: Bayley scales of infant development test II
DSM-V: Fifth edition of the diagnostic and statistical manual of mental disorders
EEG: Electroencephalogram
HCFC1: Host cell factor C1
HUMURA: Human androgen-receptor locus
IFN: Interferon
Ig: Immunoglobulin
IRAK1: Interleukin-1 receptor associated kinase 1
K-CDR: Korean-child development review
K-WAIS-IV: Korean Wechsler adult intelligence scale fourth edition
MECP2: Methyl-CpG-binding protein 2
MLPA: Multiplex ligation-dependent probe amplification
MRC: Medical research council
MRI: Magnetic resonance imaging
PCR: Polymerase chain reaction
TLR: Toll-like receptor
XCI: X chromosome inactivation
XLID: X-linked intellectual disability
