# Supplementary figures and images for: A sibship with duplication of Xq28 inherited from the mother; genomic characterization and clinical outcomes
Source: BMC Med Genet. 2017 Mar 17;18:30. doi: 10.1186/s12881-017-0394-7 (PMC5356410; doi:10.1186/s12881-017-0394-7)

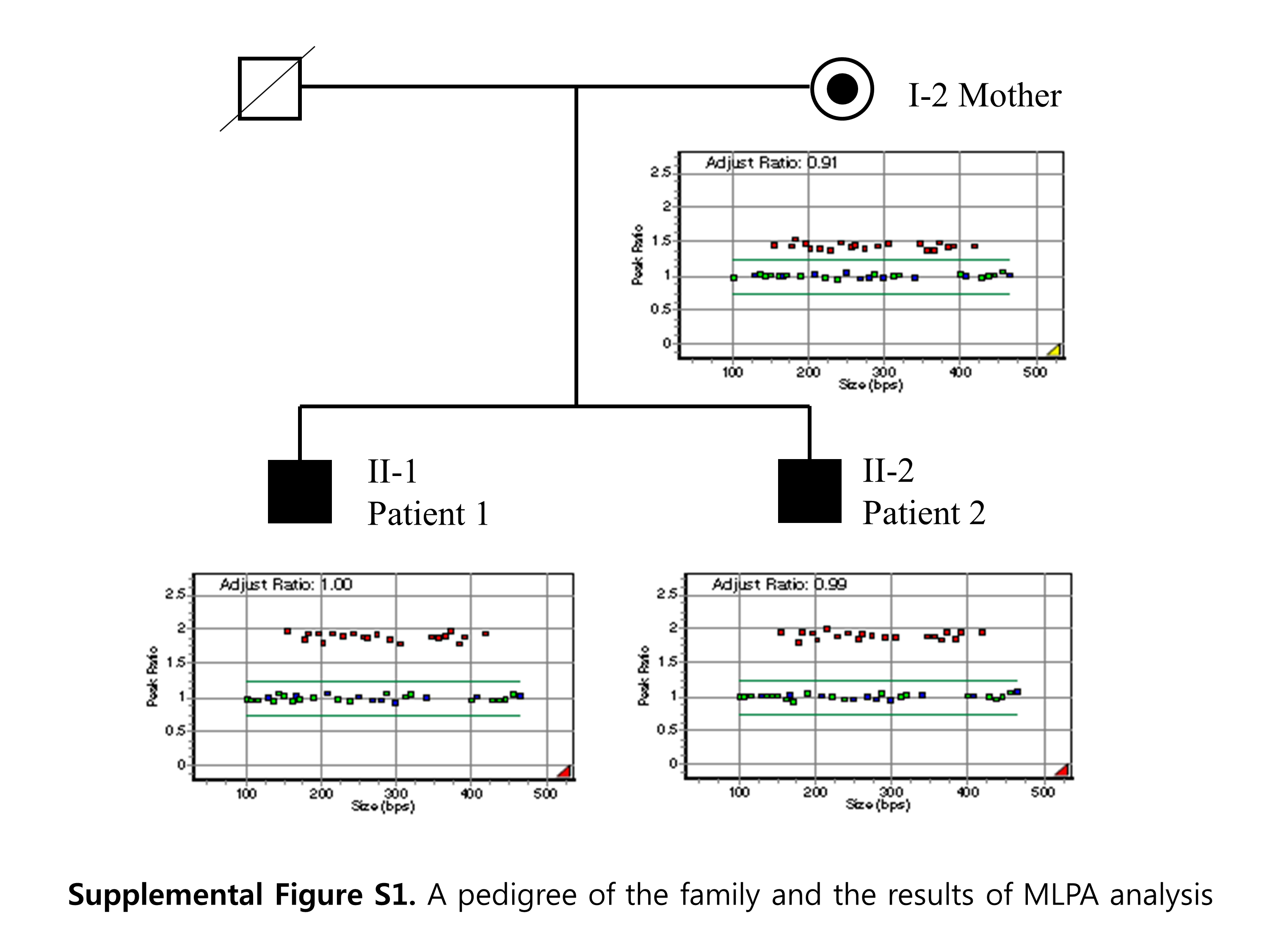

Supplement: Additional file 1: — Figure S1. A pedigree of the family and the results of MLPA analysis. (TIF 626 kb) [file 12881_2017_394_MOESM1_ESM.tif]
